# Complete mitochondrial genome of the headwater livebearer, *Poeciliopsis monacha*: the mother of clones

**DOI:** 10.1080/23802359.2016.1197066

**Published:** 2016-10-29

**Authors:** Yeon Seon Jeon, Shannon B. Johnson, Yong-Jin Won, Robert C. Vrijenhoek

**Affiliations:** a Division of EcoScience, Ewha Womans University, Seoul, South Korea;; b Monterey Bay Aquarium Research Institute, Moss Landing, CA, USA;; c Department of Life Science, Ewha Womans University, Seoul, Korea

**Keywords:** Mitochondrial genome, headwater livebearer (*Poeciliopsis monacha*), Poeciliidae

## Abstract

The sexually reproducing fish, *Poeciliopsis monacha* (Actinopterygii, Cyprinodontiformes, Poeciliidae), is the maternal ancestor of six hybrid biotypes that reproduce clonally. The gene content and order of its 16,818 bp mitochondrial genome is virtually identical with that of other sexually reproducing poeciliid fishes, providing no evidence for a mitochondrial involvement in the origins of all-female reproduction.

The headwater livebearer, *Poeciliopsis monacha* (Actinopterygii, Cyprinodontiformes, Poeciliidae), is native to the springs and arroyos of northwestern Mexico. It served as the maternal ancestor for six all-female biotypes: the gynogenetic allotriploids *P.* 2*monacha-lucida*, *P. monacha*-2*lucida*, and *P. monacha-viriosa-lucida*; and the hybridogenetic allodiploids *P. monacha-lucida*, *P. monacha*-*occidentalis*, and *P. monacha*-*latidens* (Vrijenhoek 1998). Clonal reproduction arises spontaneously in interspecific hybrids with *P. monacha* mothers (Wetherington et al. 1987). Does its mitochondrial genome contain unusual characteristics that played a role in these origins?

Herein, we report the complete mitochondrial genome of *P. monacha* and compare it with a reference genome from other eight poeciliids (Figure 1). The specimen we examined was sampled from a headwater tributary of the Río Fuerte in Sonora, Mexico (26.908449°N, 108.671743°W; altitude 344 m), a locality from which *P. monacha* females were used to conduct laboratory syntheses of hybridogenetic biotypes (Wetherington et al. 1987). Genomic DNA was sequenced by 454 GS-Flex Titanium pyrosequencing (Roche Diagnostics Corporation, Bedford, CT) long-read version on a half-plate scale by Macrogen, Inc. (Seoul, Korea).

**Figure 1.**
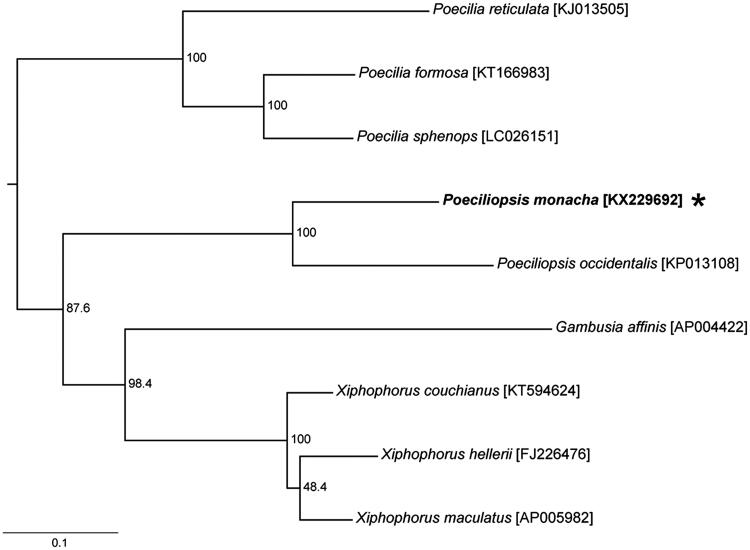
A maximum-likelihood tree of poeciliids constructed under the GTR model using the complete sequences of mitogenomes with *Xenotoca eiseni* (GenBank accession number AP006777) as an outgroup. The asterisk indicated the species in this study. Bootstrap support was shown at nodes.

We used the web-based MitoAnnotator (Kashiwa, Chiba, Japan) (Iwasaki et al. 2013) to annotate the *P. monacha* genome. Using the PhyML (Guindon & Gascuel 2003) plugin on the Geneious v.9.04 (http://www.geneious.com/), a maximum-likelihood (ML) tree was constructed under the GTR model based on the complete mitogenomic sequences of nine poeciliids and an outgroup species, *Xenotoca eiseni*, with 1000 bootstraps (Figure 1).

The *P. monacha* mitogenome examined in this study was 16,818 bp long (GenBank accession number: KX229692, a paratype specimen’s museum number at the Natural History Museum of Ewha Womans University: EWNHMFI44) with an AT-rich (55.5%) base-composition: C (29.5%) > A (28.7%) > T (26.8%) > G (15%). It contained a non-coding control region (D-loop) and 37 genes typical of vertebrates: 2 rRNA genes; 22 tRNA genes; and 13 protein-coding genes. Individual tRNA genes showed the typical cloverleaf secondary structures. The protein-coding genes showed incomplete stop codons (T–– or TA–) that presumably were completed to a TAA stop codon by post-transcriptional polyadenylation (Ojala et al. 1981). The ML tree showed that *P. monacha* has a sister relationship with the congeneric species, *P. occidentalis*.

The present analysis revealed nothing unusual in the *P. monacha* mitogenome that might contribute to the origins of the all-female alloploid biotypes. Length and AT-bias of the *P. monacha* mitogenome were similar to that of other eight poeciliids (16,533–16,771 bp, 52.2–59.1%). Gene compositions and order were identical in all nine ingroup species. Nonetheless, this result does not exclude possible interactions between specific mitochondrial gene sequences and the nuclear genome. A search for factors that promote meiotic dysgenesis in hybrids involving *P. monacha* females resumes.
